# Differences in Humoral and Cellular Vaccine Responses to SARS-CoV-2 in Kidney and Liver Transplant Recipients

**DOI:** 10.3389/fimmu.2022.853682

**Published:** 2022-04-14

**Authors:** Lucrezia Furian, Francesco Paolo Russo, Gianluigi Zaza, Patrizia Burra, Susan Hartzell, Debora Bizzaro, Marianna Di Bello, Caterina Di Bella, Erica Nuzzolese, Clara Agnolon, Sander Florman, Meenakshi Rana, Jar-How Lee, Yesl Kim, Umberto Maggiore, Jonathan S. Maltzman, Paolo Cravedi

**Affiliations:** ^1^ Department of Surgical, Oncological and Gastroenterological Sciences, Unit of Kidney and Pancreas Transplantation, University of Padua, Padua, Italy; ^2^ Multivisceral Transplant Unit-Gastroenterology, Department of Surgical Oncological and Gastroenterological Sciences, University Hospital of Padova, Padova, Italy; ^3^ Nephrology, Dialysis and Transplantation Unit, Department of Medical and Surgical Sciences, University of Foggia, Foggia, Italy; ^4^ Translational Transplant Research Center, Icahn School of Medicine at Mount Sinai, New York, NY, United States; ^5^ Recanati-Miller Transplantation Institute, Mount Sinai Hospital, New York, NY, United States; ^6^ Division of Infectious Diseases, Department of Medicine, Icahn School of Medicine at Mount Sinai, New York, NY, United States; ^7^ Terasaki Innovation Center, Los Angeles, CA, United States; ^8^ Palo Alto Veterans Institute for Research, Palo Alto, CA, United States; ^9^ Dipartimento di Medicina e Chirurgia Università di Parma, Unita’ Operativa (UO) Nefrologia, Azienda Ospedaliera-Universitaria Parma, Parma, Italy; ^10^ Department of Medicine, Stanford University School of Medicine, Palo Alto, CA, United States; ^11^ Geriatric Research Education and Clinical Center, Veteran Affairs (VA) Palo Alto Health Care System, Palo Alto, CA, United States

**Keywords:** COVID-19, antibody, T cell, variant, immunosuppression

## Abstract

The antibody and T cell responses after SARS-CoV-2 vaccination have not been formally compared between kidney and liver transplant recipients. Using a multiplex assay, we measured IgG levels against 4 epitopes of SARS-CoV-2 spike protein and nucleocapsid (NC) antigen, SARS-CoV-2 variants, and common coronaviruses in serial blood samples from 52 kidney and 50 liver transplant recipients undergoing mRNA SARS-CoV-2 vaccination. We quantified IFN-γ/IL-2 T cells reactive against SARS-CoV-2 spike protein by FluoroSpot. We used multivariable generalized linear models to adjust for the differences in immunosuppression between groups. In liver transplant recipients, IgG levels against every SARS-CoV-2 spike epitope increased significantly more than in kidney transplant recipients (MFI: 19,617 vs 6,056; P<0.001), a difference that remained significant after adjustments. Vaccine did not affect IgG levels against NC nor common coronaviruses. Elicited antibodies recognized all variants tested but at significantly lower strength than the original Wuhan strain. Anti-spike IFN-γ-producing T cells increased significantly more in liver than in kidney transplant recipients (IFN-γ-producing T cells 28 vs 11 spots/5x10^5^ cells), but this difference lost statistical significance after adjustments. SARS-CoV-2 vaccine elicits a stronger antibody response in liver than in kidney transplant recipients, a phenomenon that is not entirely explained by the different immunosuppression.

## Introduction

Since the beginning of vaccination programs against COVID-19, several populations such as immunocompromised patients have been considered as the priorities for immunization (1). Solid organ transplant (SOT) recipients elicit reduced immunogenicity to a number of vaccines, due to the inhibitory effects of antirejection drugs on T cell activation, interaction with antigen-presenting cells, and decreased B-cell memory responses (2–4).

Current evidence indicates that the immunogenicity of SARS-CoV-2 vaccine is also suboptimal in SOT recipients (5–12). Previously identified risk factors for poor vaccine immunogenicity include older age, shorter time from transplantation, use of mycophenolate mofetil (MMF) and belatacept (13), and worse allograft function (14–19).

However, a detailed understanding of the impact of anti-rejection therapies on T cell and antibody response to SARS-CoV-2 vaccine is still lacking. Another critical point to address is whether differences in response rates across recipients of various organ transplants depend on the various immunosuppressive strategies or is linked to the organ *per se*. For instance, liver transplant recipients have significantly higher serological responses (16, 18) than kidney transplant recipients (20, 21), but the two populations have not been clearly compared face-to-face in studies evaluating both cellular and antibody responses.

To address these issues, we designed a prospective study testing antibody response and viral-reactive T cells in kidney and liver transplant recipients who received mRNA-based SARS-CoV-2 vaccination. We used multiple regression analyses to dissect the role of immunosuppression versus the kind of transplanted organ in the immunological response to SARS-CoV-2 vaccine. We also tested the antibody response against the most relevant SARS-CoV-2 variants and common coronaviruses.

## Materials and Methods

### Study Population

Our study included 52 consecutive consenting adult kidney and 50 liver transplant recipients followed-up at the University Hospital of Padua-Department of Surgery, Oncology and Gastroenterology (Multivisceral Transplant Unit-Gastroenterology and Kidney and Pancreas Transplantation Unit) who received BNT162b2 (Comirnaty^®^) mRNA Covid-19 Vaccine between March and April 2021. At the time of vaccination, all patients had a negative PCR swab test for SARS-CoV-2. Blood samples were collected at three time points: before 1^st^ dose (T0), before 2^nd^ dose (T1; 3 weeks after the 1^st^ dose) and 3 weeks after 2^nd^ dose (T2).

### Safety

All patients underwent vital sign measurement and physical examination before vaccination and were then monitored for immediate adverse events (AEs) up to 30 minutes after each vaccination, including local and systemic adverse reactions. At 3 months after the first vaccine administration, patients were asked about AEs.

Participants were also encouraged to contact the transplant center to report any possible infections, especially those who developed respiratory symptoms.

### Blood Collection, Serum Isolation and Storage

Blood was collected in sterile tubes, allowed to clot, and then centrifuged to separate the serum. Samples were aliquoted and stored at -20°C until analyses. PBMCs were isolated from separate tubes (containing EDTA) by density-gradient centrifugation using Ficoll-Paque and stored in liquid nitrogen.

### Anti-SARS-CoV-2 Antibody Measurement

Detection of SARS-CoV-2 specific IgG antibodies directed against the full trimeric spike protein, the individual spike 1 (S1), spike 2 (S2), and receptor binding domains (RBD) of the spike protein, and the nucleocapsid protein (NC) and Spike S1 fragments from six other coronaviruses, namely HCoV-229E, HCoV-HKU1, HCoV-NL63, HCoV-OC43, MERS-CoV and SARS-CoV-1 was performed with the One Lambda single-antigen bead assay, [LABScreen™ COVID Plus ^®^, One Lambda], as previously described (22). Plates were then analyzed on a Luminex FLEXMAP 3D instrument (Luminex Corp. Austin, TX). Thresholds for positivity of each antibody were defined based on package insert.

### Antibodies Against SARS-CoV-2 Variants and Other Common Coronaviruses

Spike S1 and RBD fragments from various SARS-CoV-2 variants (Sino Biologicals, Wayne, PA) with the amino acid substitutions are listed in **Supplementary Table 1** and schematically in **Figure 3A**. Each variant was conjugated to separate beads that are not overlapped with the beads on the existing LABScreen™ COVID Plus ^®^ bead panel. The variant bead pool was spiked into the LABScreen™ COVID Plus ^®^ bead panel before the assay was performed.

### IFN-γ/IL-2 FluoroSpot

PBMC were seeded at 500,000 cells/well in 96 well FluoroSpot plates (Cellular Technology Ltd) with CTL-Test Media cell culture medium containing 1% L-glutamine and anti-CD28 mAb (0.1 µg/ml). Test wells were performed in duplicate and supplemented with 15-mer overlapping peptides covering the immunedominant regions of the S glycoprotein (573 amino acids) (PepTivator SARS-CoV-2 Prot S, Miltenyi Biotec), the complete NC protein (102 peptides) (PepTivator SARS-CoV-2 Prot N, Miltenyi Biotec) and the complete M protein (53 peptides) (PepTivator SARS-CoV-2 Prot M, Miltenyi Biotec) at a final concentration of 0.5 µg/ml. Negative control wells contained 20% DMSO and lacked peptides while positive control wells included CEF-MHC Class I Peptide Pool “Plus”. The fluorophore conjugates used were CTL Red-690 and FITC-520. Assays were incubated for 24 h at 37°C. The readout was performed following the manufacturer’s instructions for the Human IFN-γ/IL-2 Double-Color FluoroSpot kit and spots were counted using an automated ImmunoSpot Analyzer Professional System (both from Cellular Technology Ltd.). To quantify antigen-specific responses, spots of the negative control wells were subtracted from the mean spots test wells, and the results were expressed as IFN-γ, IL-2, or dual-expressing-producing spot forming units (SFUs) per 5 x 10^5^ PBMCs.

### Statistical Analyses

All data were analyzed using Stata 17.0 (2021, Stata Corp LP, College Station, TX) and R version 4.1.1 (R Core Team, 2021. https://www.R-project.org/). A two-sided P value < 0.05 was regarded as statistically significant unless stated otherwise. We compared baseline continuous variables between kidney and liver transplant recipients using Mann-Whitney test for continuous variables, and Fisher’s exact test for categorical variables. We used generalized estimating equations to compare at each time point the proportions of antibody vaccine responders, the MFI levels of anti-RBD antibodies, and the spot counts of IFN-γ FluoroSpot. For the subset of patients taking tacrolimus, we used generalized linear models to compare, at time point T2 only, MFI of anti-RBD antibodies and spot counts of IFN-γ FluoroSpot, before and after adjusting for induction therapy (indicator variable), tacrolimus levels, mycophenolate use (indicator variable), steroids (indicator variable). In the above mentioned multiple regression models, proportions of responders were fitted with a Bernoulli distribution, MFI levels with gamma distribution (to accommodate the non-normal distribution with long right tails of antibody levels), and spot counts of IFN-γ FluoroSpot with zero-inflated negative binomial multivariable regression (23) (the zero-inflated model accounted for excess zero counts). We used the Huber/White/sandwich estimator of variance for all the regression models. We used biplots and correlation plots from principal component analysis to summarize the main relationship between the variables in a visual manner, using the R package FactoMineR and factoextra. The Stata and R code for all the analyses is freely available at: https://github.com/UMaggiore/COVID-19-Vaccines-Liver-Kidney-Tx.

### Study Approval

The study was conducted in accordance with the Declaration of Helsinki, and the protocol was approved by the Padua IRB. Informed consent was obtained by all patients prior to participation.

## Results

### Study Population and Clinical Outcomes

The study included 102 consenting adult organ transplant recipients (52 kidney and 50 liver transplant recipients) who received a mRNA vaccine between March and April 2021 (**Table 1**). All subjects completed the two vaccination doses. Two kidney transplant recipients and zero liver transplant recipients had prior documented SARS-CoV-2 infection.

**Table 1:** Study population’s characteristics.

	Overall (n=102)		SOT Type				P value
			Kidney (n=52)		Liver (n=50)		
Age at time of the first dose (yrs)	102	60.7 ± 9.9	52	57.5 ± 10.8	50	64.0 ± 7.7	0.001
Gender (% M)		71 (69.6%)		34 (65.4%)		37 (74.0%)	0.393
Previous COVID-19		2 (2.0%)		2 (3.8%)		0	0.495
Months elapsed since transplantation	102	125.6 ± 154.1	52	110.5 ± 79.0	50	141.4 ± 204.9	0.959
BMI (Kg/m^2^)	77	25.2 ± 4.6	52	24.6 ± 4.1	25	26.3 ± 5.4	0.140
Donor type (% Living)		9 (8.8%)		9 (17.3%)		0	0.003
Induction therapy							
*No Induction*		39 (38.2%)		9 (17.3%)		30 (60.0%)	<0.001
*ATG*		30 (29.4%)		30 (57.7%)		0	
*Basiliximab*		33 (32.4%)		13 (25.0%)		20 (40.0%)	
Tacrolimus		84 (82.4%)		46 (88.5%)		38 (76.0%)	0.123
Cyclosporine		13 (12.7%)		6 (11.5%)		7 (14.0%)	0.773
mTOR-inhibitors		24 (23.5%)		9 (17.3%)		15 (30.0%)	0.164
MMF		57 (55.9%)		41 (78.8%)		16 (32.0%)	<0.001
Steroids		43 (42.2%)		40 (76.9%)		3 (6.0%)	<0.001
Tacrolimus trough levels (ng/mL)	84	5.2 ± 2.0	46	6.2 ± 1.4	38	3.9 ± 1.8	<0.001
Cyclosporine trough levels (ng/mL)	14	111.1 ± 116.9	6	80.0 ± 9.7	8	134.4 ± 154.4	1.000
mTOR-inhibitors trough levels (ng/mL)	9	4.1 ± 1.2	7	3.7 ± 0.9	2	5.6 ± 0.6	0.111
MMF dosage (mg/day)	58	834.3 ± 556.4	42	741.2 ± 298.1	16	1078.8 ± 919.5	0.349
History of treatment for rejection		5 (4.9%)		0		5 (10.0%)	0.025
Cause of ESKD							
*Glomerular disease*		9 (17.3%)		9 (17.3%)		0	
*ADPKD*		15 (28.8%)		15 (28.8%)		0	
*Other Congenital/Pyelonephritis/Interstitial nephritis*		6 (11.5%)		6 (11.5%)		0	
*Nephroangiosclerosis*		4 (7.7%)		4 (7.7%)		0	
*Systemic vasculitis*		1 (1.9%)		1 (1.9%)		0	
*Other*		17 (32.7%)		17 (32.7%)		0	
Cause of liver failure							
*Alcoholic cirrhosis*		11 (22.0%)		0		11 (22.0%)	
*HBV +/- HDV cirrhosis*		12 (24.0%)		0		12 (24.0%)	
*HCV cirrhosis*		14 (28.0%)		0		14 (28.0%)	
*HBV + HCV cirrhosis*		2 (4.0%)		0		2 (4.0%)	
*Alcoholic + HCV and/or HBV +/- HDV*		3 (6.0%)		0		3 (6.0%)	
*PBC or PSC*		5 (10.0%)		0		5 (10.0%)	
*Cryptogenic or dysmetabolic cirrhosis*		3 (6.0%)		0		3 (6.0%)	
*Previous cancer*		37 (36.3%)		18 (34.6%)		19 (38.0%)	0.837
*Autoimmune disease*		1 (1.0%)		0		1 (2.0%)	0.490

Continuous data are reported as number of nonmissing variables, mean ± SD; categorical data are reported as number of nonmissing variables and percentages. SOT, solid organ transplant; ATG, anti-thymocyte globulin; MMF, mycophenolate mofetil; ESKD, end stage kidney disease; ADPKD, autosomal dominant polycystic kidney disease; HBV, hepatitis B virus; HDV, hepatitis D virus; HCV, hepatitis C virus; PBC, primary biliary cholangitis; PSC, primary sclerosing cholangitis.

At vaccination, median time from transplantation was over 10 years, but the range was wide in both cohorts. Median age was 60.8 years and kidney transplant recipients were significantly younger than liver transplant recipients (1). Most kidney and all liver recipients obtained the organs from deceased donors.

The maintenance immunosuppression regimens included calcineurin inhibitors, mTOR inhibitors, MMF, and steroids. The use of steroids and MMF was higher in kidney transplant recipients (P<0.001 for both, **Table 1**). Unlike kidney transplant recipients, no liver transplant recipients received thymoglobulin induction, and a proportion of them received no induction treatment.

Overall, the vaccine was well tolerated. The patients were interviewed at the follow-up visit, three months after first vaccine dose, and they reported no adverse events. During the 6 months follow-up period after vaccination, 1 liver transplant recipient developed SARS-CoV-2 infection.

### Integrated Analysis of Anti-SARS-CoV-2 Vaccine Response

**Figure 1:**
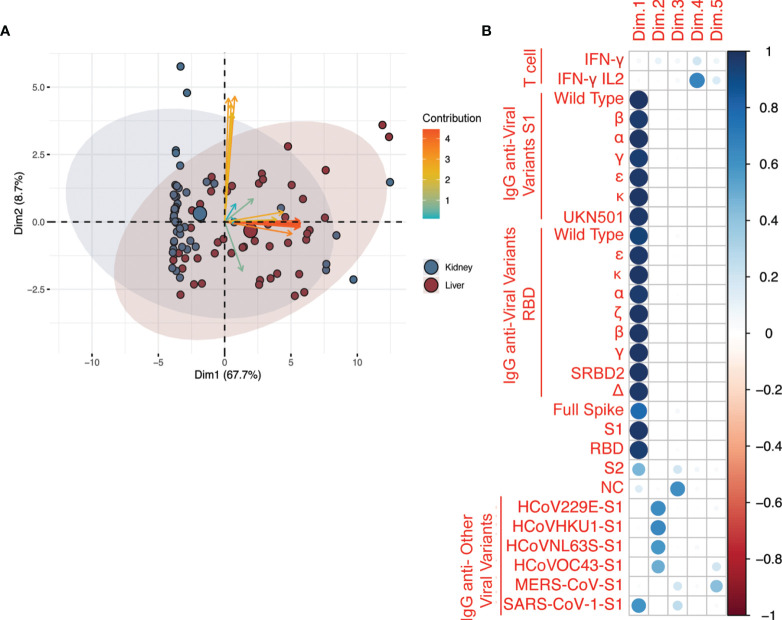
Principal component analysis (PCA) of all the assays after second vaccination. **(A)** Analysis included antibodies against anti-SARS-CoV-2, anti-SARS-CoV-2 variants, and common coronaviruses, and IFN-γ and IL-2 T cell responses. The axes represent the two variables, among all those generated by PCA, that ranked first in terms of proportion of assay variability. The variables are represented by arrows; blue arrows = Fluorospot assays, orange arrows = anti-SARS-CoV-2 serological assays, yellow = serological assays for other coronaviruses. The angle between arrows represents the correlation between assays: assays with the same direction have a correlation coefficient of 1, those with opposite directions have a correlation coefficient of -1. Those that are perpendicular to each other have a correlation coefficient of 0. The patients are represented by data points with individual point size proportional to the quality of representation in the bi-dimensional plot. The plot is based on data after two vaccinations only (T2). The categorical variable type-of-solid-organ-transplantation (i.e. kidney vs liver) is added to the plot as a supplementary variable to visualize how the pattern of correlated variables and cloud of data points are distributed between types of solid organ transplantation. The colored ellipses represent the 95% confidence ellipses of the scatter around overall assay mean of each group (liver = blue or kidney = red). **(B)** Correlation between the five principal components extracted from PCA and the original variables (assays: T cell reactivity and IgG against SARS-CoV2 antigens and variants and other coronaviruses, including common coronaviruses: HCoV229E-S1, HCoVHKU1-S1, HCoVNL63S-S1, HCoVOC43-S1). As shown by the legend in the rightmost column, the correlation is represented by a color gradient as follows: blue for positive correlation, red for negative correlation, and white for no correlation. The correlation coefficient is represented by a circle, the diameter of which is proportional to the strength of the correlation. The variability explained by the principal components Dim.1 to Dim.5 was 67.7, 8.7, 5.6, 3.8, and 3.5%, respectively (not shown).

To identify the key elements of the immune response to SARS-CoV-2 vaccine, we integrated the antibody and T cell results at T2 (3 weeks after the second vaccine administration) for all organ transplant recipients using a principal component analysis (PCA). Antibody levels against SARS-CoV-2 spike antigens (represented by the orange arrows) were the main determinant of overall variability of assay results. The contribution given to the overall variability (depicted by the length of the arrows along the x-axis in **Figure 1A**) was also related to the type of solid organ transplantation (**Figure 1A**). PCA analysis further showed that the first principal component was highly and equally correlated with all anti-SARS-CoV-2 antibodies (**Figure 1B**). Therefore, we used anti-receptor binding domain (RBD) antibody levels as surrogate marker of all other anti-SARS-CoV-2 antibody responses (including S, S1, and S2 epitopes) as these antibodies have been associated with the highest viral neutralizing capacity (24). Because PCA analysis also suggested that IgG anti-common cold coronavirus responses were uncorrelated with anti-SARS-CoV-2 responses (**Figure 1A**), we analyzed other anti-other coronaviruses antibody responses independently of anti-SARS-CoV-2 antibody responses. Finally, PCA suggested that T cell responses projected on different dimensions compared to antibody responses (**Figure 1B**). Therefore, we did not try to search for joined patterns of antibody and T cell responses, and we analyzed T cell response separately.

### Anti-SARS-CoV-2 Vaccine Antibody Response

**Figure 2:**
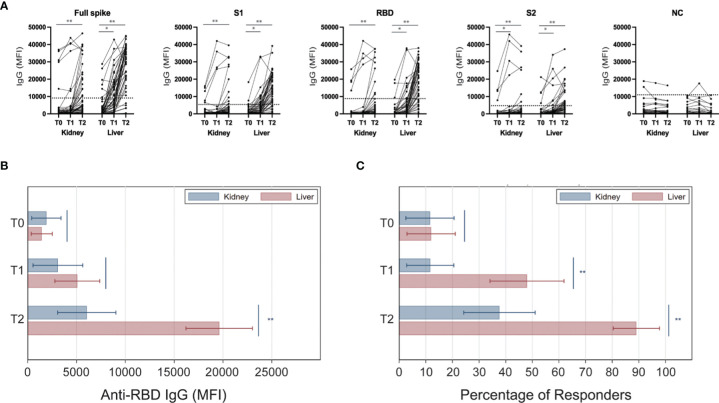
Anti-viral IgG levels in kidney and liver transplant recipients after SARS-CoV-2 vaccination. Serum samples were collected at the time of the first (T0) and the second (T1) mRNA SARS-CoV-2 vaccine administration, and at 3 weeks after the second dose (T2). **(A)** Serological response of individual subjects against each of the tested viral antigens. Level considered positive is indicated by dotted horizontal lines for each antibody specificity. **(B)** Levels of IgG anti-RBD in kidney (blue) and liver (red) kidney transplant recipients at each time point. **(C)** Analyses shown in panel B were repeated after stratifying patients based on anti-RBD antibody response (MFI threshold for positivity: 8880). Horizontal lines represent 95 percent confidence intervals. *P < 0.05, **P < 0.01. MFI, Mean Fluorescence Intensity.

We measured total IgG levels against SARS-CoV-2 trimeric spike (S), S1, S2, RBD, and nucleocapsid (NC) viral antigens at the time of the first vaccine dose (before vaccination; T0), after the first vaccine dose (at the time for the second vaccination; T1), and at 3 weeks after the second vaccine dose (T2) (**Figure 2A**). In response to vaccination, individual transplant recipients had either no response or had increased antibody levels against several epitopes of the spike protein. In contrast, anti-NC IgG levels did not significantly change after vaccination, consistent with the fact that this viral antigen is not included in the mRNA vaccine (**Figure 2A**). Based on serologies present at T0, there were five kidney and five liver transplant recipients with evidence of previous immune responses to SARS-CoV-2. This allowed us to separate visualization of longitudinal changes in antibody strength in those with or without previous infection (**Supplementary Figures 1 A, B**).

While baseline (T0) MFI levels of anti-RBD IgG did not differ between kidney and liver transplant recipients, by T2 antibodies increased significantly more in liver than in kidney transplant recipients (MFI increase: -14037 [95% CI: -9999 to -18076; P<0.001]; **Figure 2B**). Therefore, at T2, mean MFI of anti-RBD IgG was more than three times higher in liver compared to kidney transplant recipients (MFI 19617 vs 6056; P<0.001) (**Figure 2B**).

Based on the manufacturer cutoff, we next defined patients with antibody levels for anti-RBD IgG (MFI: 8800) as “responders”. Consistent with the MFI data (**Figure 2B**), the percentage of responders increased significantly less in kidney than in liver transplant recipients at both T1 and T2 (**Figure 2C**). Similar trends were seen for S, S1, and S2 epitopes (data not shown). In summary, kidney transplant recipients had an inferior serologic response compared to liver transplant recipients (38.0% vs 89.6% at T2) (**Figure 2C**). These trends did not change when we limited analysis to only patients who were non-responders at T0 (no prior immunization and no serological evidence of previous infection; data not shown). Additional sensitivity analyses of the subgroup of patients on MMF, including MMF dose and those including the variable mTORi did not change our findings.

### Crude and Adjusted Analyses of Post-Vaccine Antibody Responses Against SARS-CoV-2 Variants

**Figure 3:**
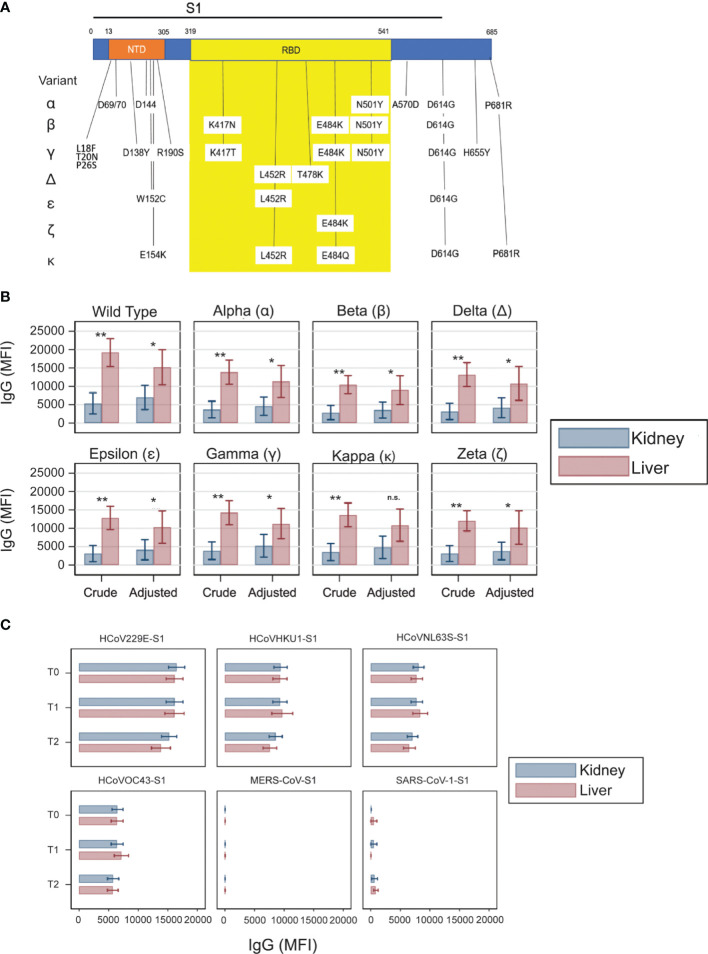
Antibody responses against the SARS-CoV-2 variants and non-SARS-CoV-2 coronaviruses. **(A)** Schematic of the SARS-CoV-2 S1 region with N terminal domain (NTD) and receptor binding domain (RBD) indicated. Vertical drop downs list the location and amino acid changes from each variant conjugated to the beads. **(B)** Crude and adjusted levels of IgG anti-RBD in various SARS-CoV-2 variants in kidney (blue) and liver (red) kidney transplant recipients on tacrolimus at T2. Adjusted analysis was adjusted for induction, blood tacrolimus levels, mycophenolate use, and steroid use. Horizontal lines represent 95 percent confidence intervals. n.s., not significant; *P < 0.05, **P < 0.01. **(C)** Antibody strength directed against the S1 region of non-SARS-CoV-2 coronaviruses. MFI thresholds for positivity: HCoV229ES1: 11,636; HCoVHKU1S1: 6,161; HCoVNL63SS1: 6,392; HCoVOC43S1: 4,888; MERSCoVS1: 27; SARSCoV1: 58.

Since liver transplant recipients were receiving lower immunosuppression compared to kidney transplant recipients, we repeated the analyses by adjusting these comparisons for induction and maintenance immunosuppression (tacrolimus levels, mycophenolate use, and steroid use). This analysis was an attempt at estimating to what extent the difference in MFI at T2 would have changed had the two groups received the same amount and type of immunosuppression. Since adjustments for tacrolimus levels can be performed only within individuals on tacrolimus therapy, we focused only on patients on tacrolimus (n = 84). Besides Ig anti-RBD, we carried out the same analysis on the antibodies against each of several other variants of SARS-CoV-2 (**Figure 3A**). Elicited antibodies recognized all variants tested, but at lower strength when compared with the original Wuhan strain (**Figure 3B**). The analyses showed that, although adjustment for immunosuppression attenuated the differences in MFI at T2 between kidney and liver transplant recipients, the difference remained substantial for virtually any SARS-CoV-2 variant (**Figure 3B**).

### Antibody Response Against Other Coronaviruses

To determine if the differences in antibody levels was specific to vaccine induced antibodies, we measured antibody levels against other coronaviruses over the course of vaccination. There was no difference in antibody levels between kidney and liver transplant recipients measured at any time point (**Figure 3C**).

### T Cell Responses

**Figure 4:**
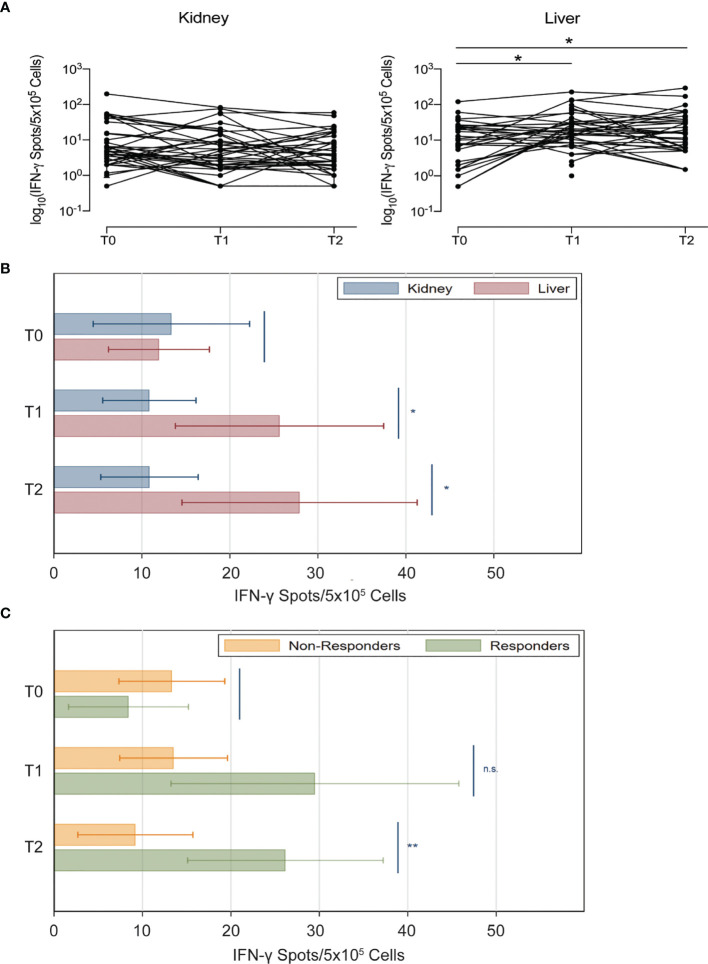
Anti-spike IFN-γ+ T cell responses in kidney and liver transplant recipients after SARS-CoV-2 vaccination. **(A)** Anti-spike IFN-γ+ T cell spots of individual subjects (natural logarithmic scale). **(B)** Number of spots of anti-S IFN-γ FluoroSpot in kidney (blue) and liver (red) transplant recipients at each time point. **(C)** Analyses shown in panel B were repeated after stratifying patients based on anti-RBD antibody response (MFI threshold for positivity: 8880) (kidney and liver transplant recipients have been pooled together for this analysis). Horizontal lines represent 95 percent confidence intervals n.s., not significant; *P < 0.05, **P < 0.01.

We measured anti-S protein IFN-γ and IL-2 T cell responses at the same time points of antibody assessment. Similar to the anti-S antibody results, we found that both kidney and liver transplant recipients had similarly few viral reactive T cells pre-vaccine (**Figure 4A**). However, after vaccination, IFN-γ producing cells did not increase in kidney transplant recipients (T1 vs T0, P=0.51; T2 vs T0, P=0.59), whereas they increased after the first dose in liver transplant recipients (T1 vs T0, P=0.010; T2 vs T0, P=0.017). Accordingly, at both T1 and T2 the number of viral reactive T cells was higher in liver compared to kidney transplant recipients (at T1, 25.6 vs 10.8 [P=0.025]; at T2, 27.9 vs 10.9 [P=0.021]; **Figure 4B**). After adjustment for immunosuppression in the subset of patients on tacrolimus, viral reactive T cells were still numerically higher in liver transplant recipients although the difference was no longer statistically significant (P=0.31; **Supplementary Figure 2**).

We next compared IFN-γ producing T cells between patients who were antibody responders and non-responders at T2 (anti-RBD IgG MFI >8800). At T2, antibody responders had a significantly greater increase in IFN-γ producing T cells than non-responders (26.2 vs 9.3; P=0.010) (**Figure 4C**).

We detected very few IL-2-producing or IL-2/IFN-γ double producing T cells in response to S protein either before or serially after vaccination (**Supplementary Figure 3**). While there is no statistical difference between the organs, there were more subjects with IL-2+ IFN-γ+ cells among the liver compared with kidney transplant recipients.

We found virtually no IFN-γ+ and IL-2+ T cells in response to M and NC proteins (not shown). Results did not change when we analyzed only patients who were non-responders at T0 (no prior immunization; data not shown).

## Discussion

To the best or our knowledge, no study has formally compared anti-SARS-CoV-2 vaccine responses between kidney and liver transplant recipients, but comparisons across separate studies indicate that the rates of response are higher in liver transplant recipients (10, 16, 25). Consistent with previous studies, we found that liver transplant recipients have higher antibody responses against SARS-CoV-2 S antigen upon vaccination than kidney transplant recipients. This was associated with the more common use of thymoglobulin induction, higher use of MMF, the higher blood trough levels of tacrolimus in kidney transplant recipients. However, differences in antibody response persisted also after adjusting for all these factors, suggesting that there is an independent effect of the transplanted graft on the antibody response. Intriguingly, kidney transplant recipients had lower seroconversion despite being younger than liver transplant recipients. As younger age has been associated with higher response rates than older age (26, 27), the difference in seroconversion might have been even more pronounced if patients in the two groups had similar age.

With the limitations of a relatively small sample size, our data support the hypothesis that poor kidney function (either pre-transplant, while patients are on dialysis or after transplant) might affect the immune response to vaccination. In particular, data indicate that uremia associates with increased T cell exhaustion (28) and low response to vaccines (29), which could, at least partially, explain, the impaired serological responses to SARS-CoV-2 vaccines in kidney transplant recipients, as well as in patients with kidney failure (30, 31). Consistent with this hypothesis, response to SARS-CoV-2 vaccine in patients with liver failure is not significantly impaired (32).

Driven by the results of the PCA analysis, we decided to use anti-RBD IgG responses as a surrogate of the other antibodies. These antibodies have been used by most prior publications (8, 33), which allows us to put our data in the prospective of the available literature. However, our study contains additional granular information on the IgG levels against multiple epitopes in the S protein. These data indicate that, although post-vaccine anti-spike protein IgG levels were lower in transplant patients than what has been previously reported in the general population, transplant recipients have no selective impairment in the response against multiple defined epitopes of the viral protein.

Our study also tested the vaccine induced serological responses of organ transplant recipients against multiple variants of the SARS-CoV-2. While direct comparisons across different epitopes is not possible due to the potential for different numbers of antigen molecules of the Luminex beads used for testing, our data indicate that transplant recipients showing a serological response to the Wuhan variant of the virus also produce antibodies against the other virus variants. Importantly, all the tested variants include changes in the sequence of the RBD portion of S protein (**Figure 3A**) which may affect their antigenic properties. This finding is important, especially considering the growing number of variants that are emerging. Intriguingly, liver transplant recipients had higher IgG levels against many of these variants than kidney transplant recipients even after statistical adjustments. Further studies are needed to confirm this potentially relevant finding.

We also used Luminex to test antibody responses against common coronaviruses and, as predicted, we found that these responses were not affected by vaccination, supporting the specificity of our assays.

We found a relationship between anti-SARS-CoV-2 antibody formation and T cell responses, as documented by the fact that patients with antibody seroconversion (responders) had also higher levels of IFN-γ producing T cells at T2 (**Figure 4C**). These data confirm and expand previous findings by others (34, 35). However, we also noticed patients with serological conversion that did not have detectable anti-SARS-CoV-2 IFN-γ T cell responses. This is consistent with the notion that anti-SARS-CoV-2 antibody production is, at least in part, T cell-independent (36).

Intriguingly, anti-SARS-CoV-2 IFN-γ T cell responses were more affected by immunosuppression than the anti-SARS-CoV-2 antibody formation. The significantly higher IFN-γ T responses in liver compared to kidney transplant recipients was no longer present after adjustments for immunosuppression. Although T cells are thought to represent the major contributors to IFN-γ production in response to SARS-CoV-2 antigens (37), the FluoroSpot assay does not allow discrimination of whether other cell subsets, including NK cells, are implicated in this response. The impact of the kind of transplanted graft on these cells might be minor, while they could be significantly affected by immunosuppression.

Contrary to prior studies (33, 38), we found limited numbers of anti-SARS-CoV-2 IL-2+ T cells in both kidney and liver transplant recipients. The fact that T cell response in these patients was predominantly IFN-γ+ might be due the fact that, at variance from prior studies, ours focused on early time-points, which could be dominated by IFN-γ T cell responses.

Our assays do not provide information on the specific T cell epitopes eliciting immune responses or HLA restriction nor on the T cell killing capacity. However, testing peptides with HLA restrictions would limit power of the study as the number of patients with shared HLA is limited.

We also acknowledge that, despite our multivariable analyses (including induction therapy and ongoing chronic immunosuppression), our results could be biased by confounders that we could not take into account. However, time post-transplant (which is correlated with overall exposure to immunosuppression) was similar between groups, suggesting that different immunosuppression levels between the two cohorts were not responsible for the differences in vaccine responses.

Finally, our study did not include a cohort of healthy controls. However, the literature is very clear in indicating that over 95% of healthy individuals have an effective Ab response to SARS-CoV2 vaccine (39–42). Therefore, it is reasonable to assume that the transplanted individuals included in our study had a significantly lower response regardless from the transplanted organs.

In summary, the anti-SARS-CoV-2 IgG production in response to mRNA vaccine is impaired in kidney transplant recipients more than in liver transplant recipients. This difference is largely explained by the higher immunosuppression levels in kidney transplant recipients, but it is also independently affected by the kind of transplanted organ (or failure or the native organs). Understanding the mechanisms responsible for these differences may allow unraveling new strategies to increase post-vaccine antibody production in poorly responsive individuals.

## Data Availability Statement

The raw data supporting the conclusions of this article will be made available by the authors, without undue reservation.

## Ethics Statement

The study was conducted in accordance with the Declaration of Helsinki, and the protocol was approved by the Padua IRB. Informed consent was obtained by all patients prior to participation. The patients/participants provided their written informed consent to participate in this study.

## Author Contributions

LF, FPR, PB, GZ, JM, and PC designed the study. LF, FPR, and PB collected the samples and clinical information. DB processed the clinical samples. JL and YK performed the antibody analyses by Luminex. SH performed the FluoroSpot assays. UM analyzed the data and helped writing the manuscript. JSM and PC wrote the first draft of the manuscript. All authors contributed to the article and approved the submitted version.

## Funding

This work was funded in part by Thermo Fisher Scientific/One Lambda, Inc. (to JM). PC has been supported by the NIH grant R01 AI132949. The funders were not involved in the study design, collection, analysis, interpretation of data, the writing of this article or the decision to submit it for publication.

## Conflict of Interest

JM has received research support and honoraria from Thermo Fisher Scientific/One Lambda, Inc.

The remaining authors declare that the research was conducted in the absence of any commercial or financial relationships that could be construed as a potential conflict of interest.

## Publisher’s Note

All claims expressed in this article are solely those of the authors and do not necessarily represent those of their affiliated organizations, or those of the publisher, the editors and the reviewers. Any product that may be evaluated in this article, or claim that may be made by its manufacturer, is not guaranteed or endorsed by the publisher.
