# Supplementary material for: Differences in Humoral and Cellular Vaccine Responses to SARS-CoV-2 in Kidney and Liver Transplant Recipients
Source: Front Immunol. 2022 Apr 14;13:853682. doi: 10.3389/fimmu.2022.853682 (PMC9047689; doi:10.3389/fimmu.2022.853682)
Supplement: Supplementary file 1 [file DataSheet_1.docx]

**SUPPLEMENTARY MATERIAL**

### Supplementary Table 1. SARS-CoV-2 variants and amino acid substitutions.

| Spike Fragment | WHO Label | Catalog Number | Amino Acid Differences from the Original B Lineage |
| --- | --- | --- | --- |
| S1 | N/A | 40591-V08H | (Lineage B) |
| S1 | Beta | 40591-V08H10 | **K417N, E484K, N501Y,** D614G |
| S1 | Alpha | 40591-V08H12 | HV69-70 deletion, Y144 deletion, **N501Y**, A570D, D614G, P681H |
| S1 | Gamma | 40591-V08H14 | L18F, T20N, P26S, D138Y, R190S, **K417T, E484K, N501Y**, D614G, H655Y |
| S1 | Beta | 40591-V08H15 | L18F, D80A, D215G, LAL242-244 deletion, R246I, **K417N, E484K, N501Y**, D614G |
| S1 | Epsilon | 40591-V08H17 | W152C, **L452R**, D614G |
| S1 | Kappa | 40591-V08H19 | E154K, **L452R, E484Q**, D614G, P681R |
| S1 | (Alpha) | 40591-V08H7 | HV69-70 deletion, **N501Y**, D614G |
| RBD | N/A | 40592-V08H | (Lineage B) |
| RBD | Epsilon | 40592-V08H28 | **L452R** |
| RBD | Kappa | 40592-V08H81 | **E484Q** |
| RBD | Alpha | 40592-V08H82 | **N501Y** |
| RBD | Zeta | 40592-V08H84 | **E484K** |
| RBD | Beta | 40592-V08H85 | **K417N, E484K, N501Y** |
| RBD | Gamma | 40592-V08H86 | **K417T, E484K, N501Y** |
| RBD | Kappa | 40592-V08H88 | **L452R, E484Q** |
| RBD | Delta | 40592-V08H90 | **L452R, T478K** |
| RBD | (Beta) | 40592-V08H59 | **K417N** |

### Substitutions in the RBD are high-lighted in bold.


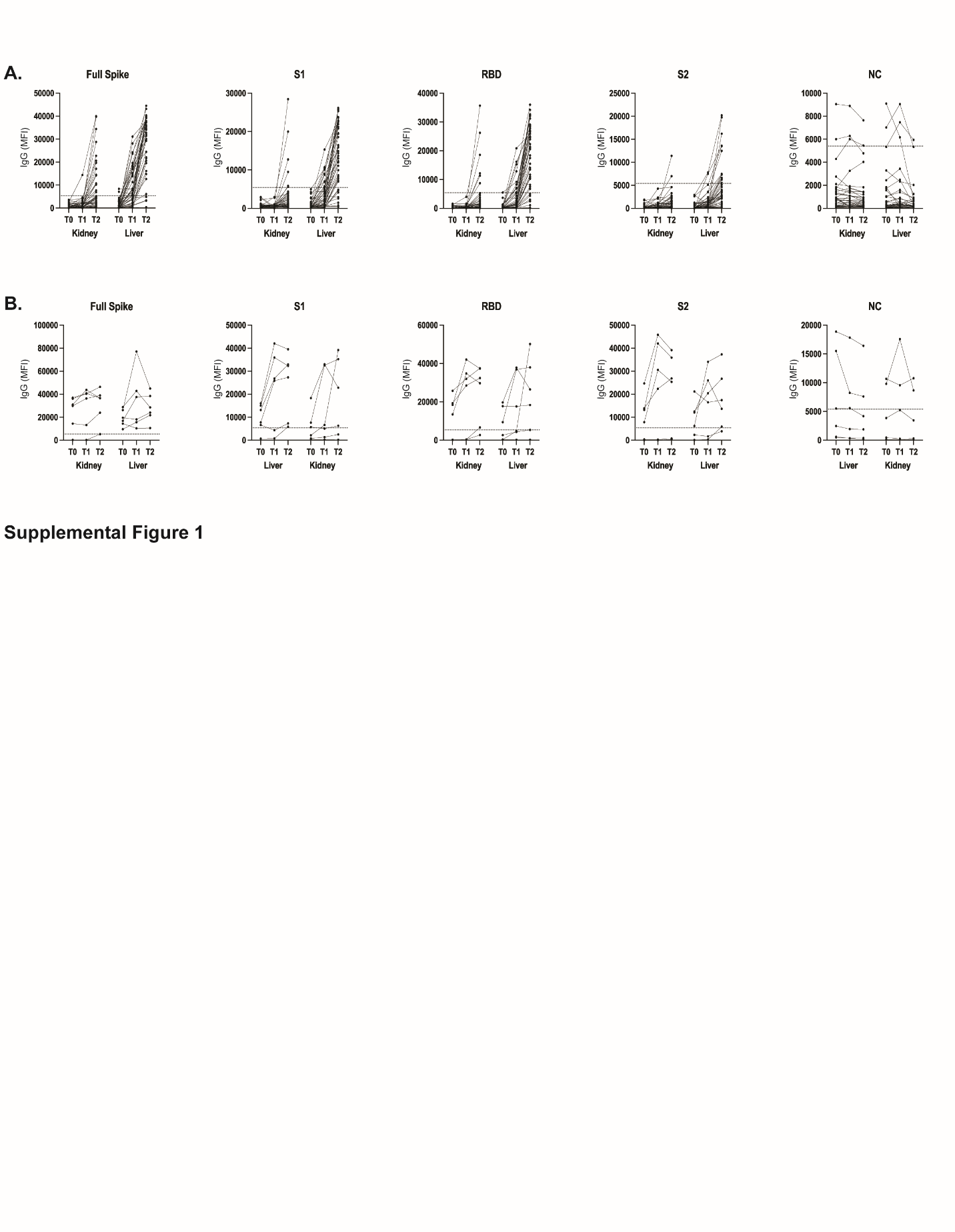


**Supplementary Figure 1. Anti-viral IgG MFI levels in kidney and liver transplant recipients after SARS-CoV-2 vaccination stratified by the presence of prior anti-SARS-CoV-2 antibodies.** Serum samples were collected at the time of the first (T0) and the second (T1) mRNA SARS-CoV-2 vaccine administration, and at 3 weeks after the second dose (T2). The figure shows serological response of individual subjects against each of the tested viral antigens. The graphs on top (panel **A**) include individuals that, at T0, showed no IgG against any of the spike protein antigens. The graphs at the bottom (panel **B**) include individuals that, at T0, showed IgG levels above the threshold of positivity for at least one of the spike protein antigens.


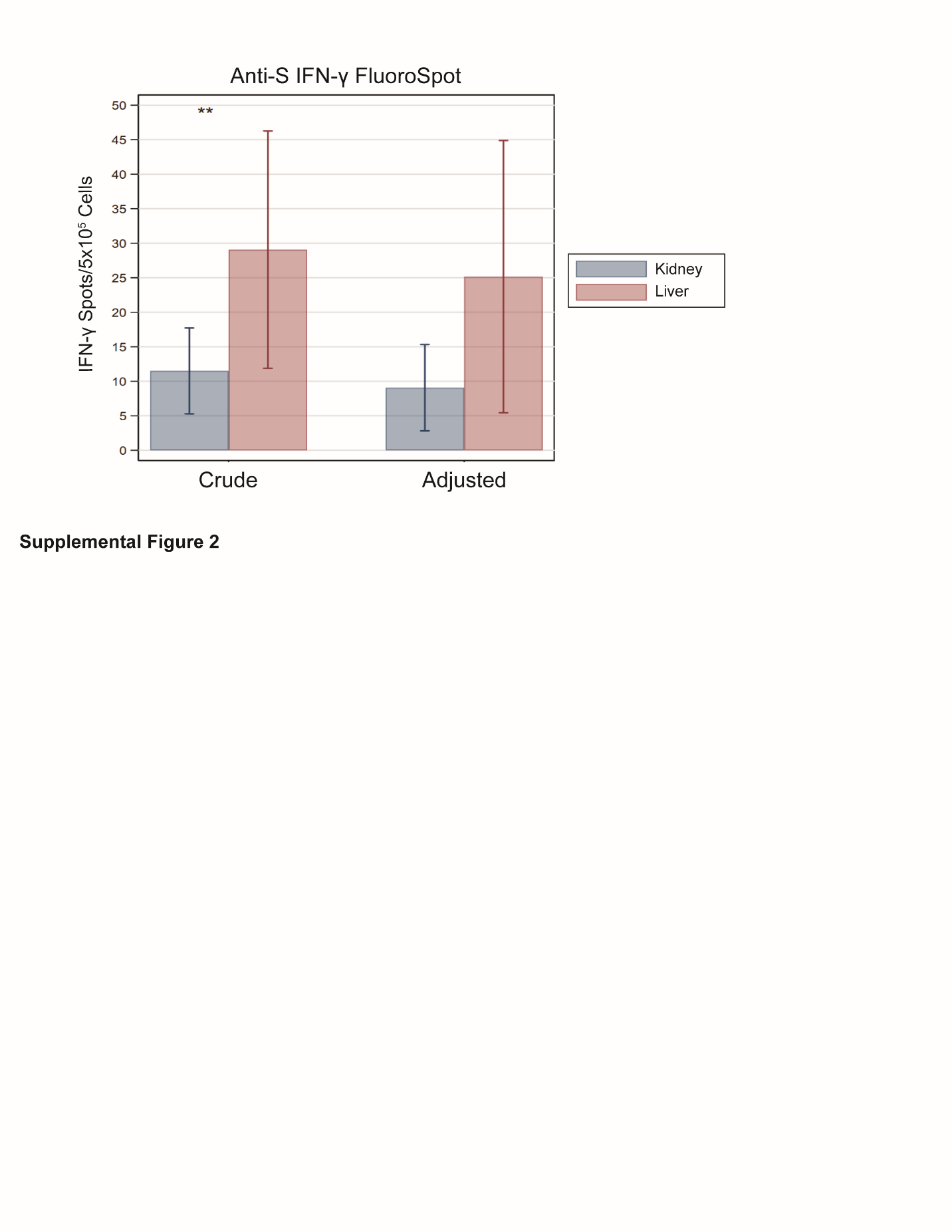


**Supplementary Figure 2.** **Crude and adjusted analyses of anti-spike IFN-γ+ T cell responses in kidney and liver transplant recipients after SARS-CoV-2 vaccination.** Crude and adjusted number of spots of anti-S IFN-γ-ELISPOT in kidney (blue) and liver (red) transplant recipients at 3 weeks after the second vaccine administration (T2). Bars represent medians and vertical lines represent 95 percent confidence intervals. *P<0.01.


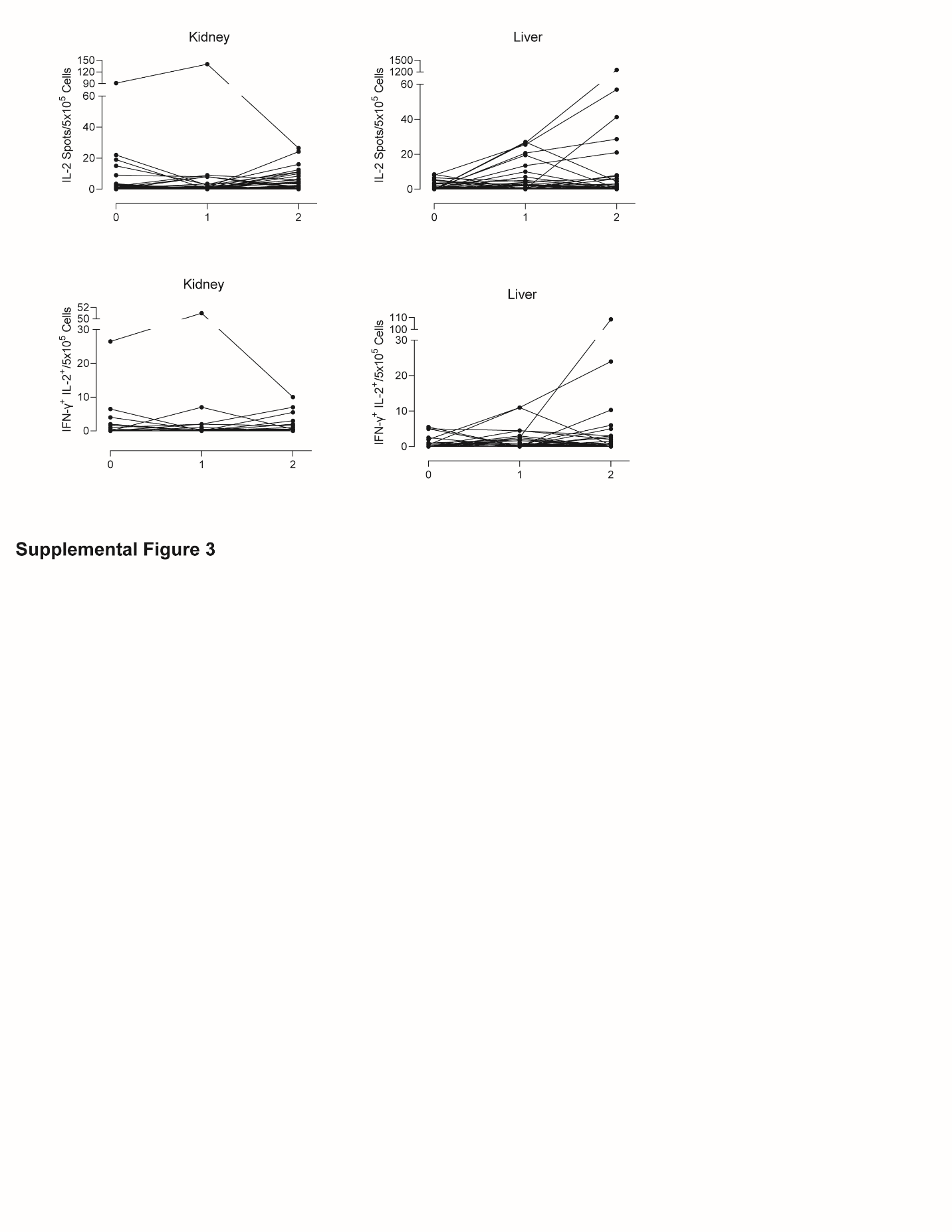


**Supplementary Figure 3.** **Anti-spike IL-2+ and IFN-γ+IL-2+ T cell responses in kidney and liver transplant recipients after SARS-CoV-2 vaccination.** Anti-spike IL-2+ (top) and IFN-γ+IL-2+ (bottom) T cell spots of individual kidney and liver transplant recipients.
